# Serum-Derived Neuronal Exosomal microRNAs as Stress-Related Biomarkers in an Atopic Dermatitis Model

**DOI:** 10.3390/biomedicines9121764

**Published:** 2021-11-25

**Authors:** Minkyoung Sung, Soo-Eun Sung, Kyung-Ku Kang, Joo-Hee Choi, Sijoon Lee, KilSoo Kim, Ju-Hyeon Lim, Gun Woo Lee, Hyo-Deog Rim, Seunghee Won, Byung-Soo Kim, Kyungmin Kim, Seoyoung Jang, Sang Gyu Kwak, Jungmin Woo, Min-Soo Seo

**Affiliations:** 1 Laboratory Animal Center, Daegu-Gyeongbuk Medical Innovation Foundation (DGMIF), Daegu 41061, Korea; tjdalsrud27@naver.com (M.S.); sesung@dgmif.re.kr (S.-E.S.); kangkk@dgmif.re.kr (K.-K.K.); cjh522@dgmif.re.kr (J.-H.C.); sjlee1013@dgmif.re.kr (S.L.); kskim728@knu.ac.kr (K.K.); 2 Department of Psychiatry, School of Medicine, Kyungpook National University, Daegu 41944, Korea; hdrim@knu.ac.kr (H.-D.R.); wonsh864@knu.ac.kr (S.W.); because99@hanmail.net (B.-S.K.); forbluewish@hanmail.net (K.K.); seoyoung870314@daum.net (S.J.); 3 College of Veterinary Medicine, Kyungpook National University, 80 Daehakro, Buk-gu, Daegu 41566, Korea; 4 New Drug Development Center, Osong Medical Innovation Foundation, Cheongju 28160, Korea; globaljh2019@gmail.com; 5 Department of Orthopedic Surgery, Yeungnam University Medical Center, Yeungnam University College of Medicine, 170 Hyonchung-ro, Namgu, Daegu 42415, Korea; gwlee1871@gmail.com; 6 Department of Medical Statistics, School of Medicine, Catholic University of Daegu, Daegu 42472, Korea; sgkwak@cu.ac.kr

**Keywords:** atopic dermatitis, stress, exosomes, miRNA, biomarkers

## Abstract

Chronic allergic inflammatory skin disease—atopic dermatitis (AD)—is characterized by eczema, pruritus, xeroderma, and lichenification. Psychological stress is one cause of this disease; however, psychological stress will also result from the presence of AD symptoms. Previous studies have shown that psychological stress triggers neuroinflammation in the brain, where microRNAs (miRNAs) in the neuronal exosomes (nEVs) were analyzed to identify the composition of the miRNAs in the nEVs and how they were altered by AD. In this study, the AD model was induced by treatment with 2,4-dinitrochlorobenzene (DNCB). The expression patterns of neuroinflammation markers, such as brain-derived neurotrophic factor, cyclooxygenase-2, and glial fibrillary acidic protein, were subsequently evaluated over time. Among these groups, there was a significant difference in DNCB 14 days expression compared with the control; therefore, nEVs were isolated from serum and next-generation sequencing was performed. The results demonstrate that 9 miRNAs were upregulated and 16 were downregulated in the DNCB 14 days compared with the control. Previous studies have shown that some of these miRNAs are associated with stress and stress-induced depression, which suggests that the miRNAs in nEVs may also be stress-related biomarkers.

## 1. Introduction

Atopic dermatitis (AD) is a chronic relapsing allergic skin inflammatory disease. AD has increased 2–3-fold in developed countries [1,2]. In addition, 15–20% of children worldwide are affected by AD, and in 1–3% of AD cases, they persist into adulthood [2]. The characteristic symptoms of AD are eczema, pruritus, xeroderma, and lichenification [1]. Various factors cause AD, such as genetic, environmental, immune, and psychological factors [3]. Among these factors, psychological stress induces an allergic response through the activation of T helper 2 (Th2) cells by activating the hypothalamic–pituitary–adrenal axis (HPA axis)—a neuroendocrine system that regulates the stress response [3]. Furthermore, the secretion of a variety of cytokines released by the immune cells is activated, such as IL-4, IL-5, IL-13, etc., which will activate an allergic response [4]. Stress is a natural response in AD patients due to the main symptoms of dermatitis [3,4]. As dermatitis symptoms can likely cause stress, psychotherapy has been provided to patients with AD to reduce stress and anxiety, whereby studies have shown that psychotherapy did indeed alleviate AD symptoms [5]. From these previous findings, the closely stress-related AD animal model was selected as a stress-related animal model.

Stress is the physical and psychological tension that occurs in situations that threaten homeostasis [6]. A stress response is initiated by the HPA axis when an individual is exposed to stress. Glucocorticoid hormones that are the final products of the HPA axis are currently of interest as stress-related biomarkers [7]. However, these hormones are not suitable for use in quantitative stress analysis as they are affected by several factors, such as circadian rhythm, sex, aging, and stressors [8,9,10,11]. In addition to the method of measuring glucocorticoid hormones, interviews and questionnaires are also conducted for the diagnosis of stress [12]. However, these methods also have limitations in quantitatively confirming stress levels. Therefore, to accurately determine and quantify stress, it is necessary to identify a suitable biomarker.

Exosomes are a subtype of extracellular vesicles, secreted through multivesicular endosome fusion with the plasma membrane. They are typically vesicles of 30–200 nm in size, and shaped as a spherical phospholipid bilayer [13,14]. They are stable in almost all body fluids, including blood, saliva, and cerebrospinal fluid, and contain intracellular components, such as proteins, lipid, ribonucleic acid (RNA), and microRNAs (miRNAs) [15]. They can also pass through the blood–brain barrier in both directions and are known to be involved in cell–cell interactions [16]. Because of these characteristics, exosomes have been actively studied as biomarkers or therapeutic targets for many diseases, such as cancer, inflammation, Parkinson’s disease, and Alzheimer’s disease [17,18,19,20]. Exosomes have also been previously shown to be associated with neuron-related regulation [21].

Among the contents of exosomes, miRNAs are small (17–24 nt) noncoding RNAs acting as posttranscriptional regulators that bind to the 3′ untranslated region of the target mRNA [22]. These miRNAs have been studied as biomarkers in a variety of diseases, such as breast cancer tumors, major depressive disorders, schizophrenia, Parkinson’s disease, and Alzheimer’s disease [23,24,25,26].

Furthermore, previous studies have shown that psychological stress is associated with neuroinflammation [27]. For example, several studies have demonstrated that an increase in glial fibrillary acidic protein (GFAP)—a marker of neuroinflammation—occurs when repeated immobilization stress or acute stress is induced in rodents [28,29]. Additionally, other research has shown that chronic stress can lead to spinal neuroinflammation, resulting in sensory hypersensitivity [30]. In this study, neuronal exosomes (nEVs) were isolated from the serum of AD animal models, and the expression patterns of neuronal exosomal miRNAs were compared to those of normal animals where an assumption of stress caused by AD affects the brain. Our findings suggest that neuronal exosomal miRNAs can be utilized as stress-specific biomarkers.

## 2. Materials and Methods

### 2.1. Animals

The experiments were performed with 8-week-old male BALB/c mice that were housed under controlled temperature, humidity, and light conditions (24 ± 2 °C, 50 ± 20% relative humidity, 12/12 h light/dark cycle, with lights on at 07:00). Additionally, animals had ad libitum access to food and water. The animal experimental protocols were reviewed and approved by the Institutional Animal Care and Use Committee of The Laboratory Animal Center of the DGMIF (IACUC; approval No. DGMIF-21011401-02; approved on 14 January 2021; Daegu, Korea), and were in accordance with their guidelines.

### 2.2. Atopic Dermatitis Animal Model Induced by 2,4-Dinitrochlorobenzene

The AD model described by Yang et al. was used [31]. In this protocol, 2,4-dinitrochlorobenzene (DNCB) (Sigma, St. Louis, MO, USA) was used to induce the AD animal model. To begin, mice were subdivided into control, DNCB 2 days, DNCB 6 days, and DNCB 14 days groups, and 5 mice were used each. The dorsal hair was then shaved, and the mice in the DNCB 2 days group were treated once with 200 μL of 1% DNCB diluted in acetone and olive oil (4:1) and sacrificed 24 h later. In the DNCB 6 days group, mice were treated once a day on days 0 and 3 with 200 μL of 1% DNCB, and sacrificed on day 6. Finally, mice in the DNCB 14 days group were sensitized with 200 μL of 1% DNCB on days 0 and 3, then treated with 0.2% DNCB daily from day 6 to day 13, and then subsequently sacrificed on day 14.

### 2.3. Histopathology to Confirm Atopic Dermatitis-like Skin Lesions

The dorsal skin tissues were fixed in 10% neutral buffered formalin and embedded in paraffin. Paraffin blocks were sectioned at 4 μm thickness and stained with hematoxylin and eosin (H&E) and toluidine blue. H&E staining was performed using Dako CoverStainer (Agilent, Santa Clara, CA, USA). Toluidine blue staining was conducted with toluidine blue solution (ScyTek Laboratories, Logan, UT, USA) according to the manufacturer’s instructions. Scanning of skin sections was performed using a Pannoramic SCAN II slide scanner (3DHISTECH Kft., Budapest, Hungary). Photomicrograph capture and epidermal thickness measurement were performed using CaseViewer (3DHISTECH Kft., Budapest, Hungary).

### 2.4. Histopathology for the Identification of Neuroinflammation in the Hippocampus

Brains were dissected and fixed in 10% neutral buffered formalin and then processed and embedded in paraffin. Sections were cut to a 4 μm thickness capturing the hippocampus. H&E staining was conducted using Dako CoverStainer (Agilent, Santa Clara, CA, USA). Immunohistochemistry (IHC) was performed to confirm the expression of neuroinflammation marker proteins. The brain sections were stained with primary antibodies for brain-derived neurotrophic factor (BDNF), cyclooxygenase-2 (COX2), and GFAP (1:500; Abcam, Cambridge, UK). Next, the sections were immunohistochemically stained with the labeled polymer Dako EnVision™+ System-horseradish peroxidase (HRP) (Agilent, Santa Clara, CA, USA) according to the manufacturer’s instructions. After staining, the brain sections were scanned using a Pannoramic SCAN II (3DHISTECH Kft., Budapest, Hungary). Photomicrographs were captured by CaseViewer (3DHISTECH Kft., Budapest, Hungary). Finally, neuroinflammation markers in the hippocampus were quantified by the ImageJ software (NIH, Bethesda, MD, USA).

### 2.5. Isolation of Exosomes from Serum

Total exosomes (tEVs) were isolated from serum using ExoQuick solution (System Bioscience, Palo Alto, CA, USA) with some modifications to the manufacturer’s instructions. Briefly, serum was centrifuged at 3500× *g* for 15 min at 4 °C to remove cell debris. Next, the debris-cleared serum and ExoQuick solution were mixed and centrifuged at 3500× *g* for 10 min. The subsequent pellet was resuspended in phosphate-buffered saline (PBS).

### 2.6. Separation of Neuronal Exosomes from Total Exosomes Isolated from Serum

The nEVs were isolated as described by Mustapic et al., with some modifications in this study [32]. Briefly, tEV samples containing PBS were incubated with anti L1 cell adhesion molecule (L1CAM; CD171) antibody (Bioss Antibodies, Beijing, China) for 1 h at 4 °C on a rotating mixer. Following the addition of Pierce^TM^ Streptavidin Plus Ultralink™ Resin (Thermo Fisher Scientific, Waltham, MA, USA) and PBS, the mixture was incubated for 1 h at 4 °C on a rotating mixer and then centrifuged at 500× *g* for 10 min at 4 °C. The supernatants were removed, and the pellets were resuspended in 0.1 M glycine-HCl (Biosesang, Seongnam, Korea). Following the mixing and vortexing, the samples were pelleted by centrifugation at 4500× *g* for 10 min at 4 °C. The supernatants were transferred to fresh tubes, and Tris-HCl (Biosesang, Seongnam, Korea) and PBS were added.

### 2.7. Transmission Electron Microscopy (TEM)

Exosomes that were isolated from serum were resuspended in cold distilled water. Exosome suspensions were loaded on formvar carbon-coated grids (Ted Pella, Inc., Redding, CA, USA) and fixed in 2% paraformaldehyde for 10 min. The solution was subsequently removed, and the samples were dried. Grids were recorded using Bio-TEM Hitachi HT7700 (Hitachi, Chiyoda, Tokyo, Japan).

### 2.8. Nanoparticle Tracking Analysis (NTA)

NTA was performed using a PMX120 (Particle Metrix, Meerbusch, Germany) NanoSight instrument in accordance with the manufacturer’s instructions.

### 2.9. Flow Cytometry

tEVs was incubated with aldehyde/sulfate latex beads (Invitrogen, Carlsbad, CA, USA) for 15 min at room temperature (RT). PBS supplemented with 3% BSA was then added, and the samples were incubated overnight on a rotating mixer. The bead-coupled exosomes were centrifuged at 3000× *g* for 10 min and washed with PBS. After washing, the samples were further centrifuged at 3000× *g* for 10 min. The supernatants were discarded, and the pellets were resuspended in PBS containing antiCD9 and antiCD81 antibodies (BioLegend, San Diego, CA, USA) for 1 h at RT. Samples were centrifuged at 3000× *g* for 10 min. The pellets were resuspended in PBS. Exosome markers were detected using flow cytometry (Galios, Beckman Coulter, Brea, CA, USA), and the analysis was performed with Kaluza software (Beckman Coulter, Brea, CA, USA).

### 2.10. Western Blot

Each tEV and nEV sample isolated from the serum was lysed using M-PER and Halt™ Protease and Phosphatase Inhibitor Cocktail (Thermo Fisher Scientific, Waltham, MA, USA). The concentration of the exosome lysate was measured using the Pierce™ BCA Protein Assay Kit (Thermo Fisher Scientific, Waltham, MA, USA) according to the manufacturer’s instructions. Next, 20 μg of exosome lysates were separated on Bolt™ 4–12% Bis-Tris Plus Gels (Invitrogen, Carlsbad, CA, USA) and transferred onto PVDF membranes (Invitrogen, Carlsbad, CA, USA). The membranes were blocked with TBS-T supplemented with 5% skim milk for 1 h at RT. After blocking, the membranes were incubated at 4 °C overnight with the primary antibodies tumor susceptibility gene 101 (TSG101; Novus Bio, Littleton, CO, USA), CD171, β-actin (Santa Cruz Biotechnology Inc., Santa Cruz, CA, USA), neuron-specific class III β-tubulin (TUJ1), neuronal nuclei (NeuN), and neuron-specific enolase (NSE) (Abcam, Cambridge, UK) (1:1000). After washing, the membranes were incubated with HRP-conjugated secondary antibody (1:2000) for 1 h at RT and washed with TBS-T. Finally, the protein bands were detected with EzWestLumi Plus (ATTO, Tokyo, Japan) and analyzed using ImageQuant™ LAS 4000 (GE Healthcare, Buckinghamshire, UK).

### 2.11. Next-Generation Sequencing (NGS) to Analyze the Differential Expression of Neuronal Exosomal MiRNAs

NGS samples were prepared by pooling nEVs from each control group and the DNCB 14 days group, which showed the greatest changes in the expression of neuroinflammation markers in the hippocampus. Then, exosomal smRNA isolation and library preparation were conducted by Macrogen (Seoul, Korea), using the SMARTer smRNA-Seq Kit (Clontech Laboratories, Inc., Mountain View, CA, USA) according to the manufacturer’s instructions. Then, miRNA sequencing was performed by Macrogen, using the HiSeq 2500 System following the HiSeq 2500 System (User Guide Document #15035786 v02 HCS 2.2.70). Differentially expressed miRNAs were identified with a threshold *p* < 0.05.

Gene ontology (GO) analysis was performed to analyze the functional enrichment of differentially expressed miRNAs, and Kyoto Encyclopedia of Genes and Genomes (KEGG) pathway analysis was performed to identify significantly enriched signaling pathways. Target genes for miRNAs showing significant changes in expression by AD were predicted using mirWalk, and all analyses of the predicted genes were performed using the database for annotation, visualization, and integrated discovery (DAVID) v6.8. *** *p* < 0.001 was applied as the criterion.

### 2.12. Statistical Analysis

All statistical analyses were performed using Student’s *t*-test. Data were considered statistically significant with a *p* value of <0.05. The statistical data were analyzed in Microsoft Excel software.

## 3. Results

### 3.1. Confirmation That Atopic Dermatitis Is Caused by 2,4-Dinitrochlorobenzene in the SKIN

**Figure 1:**
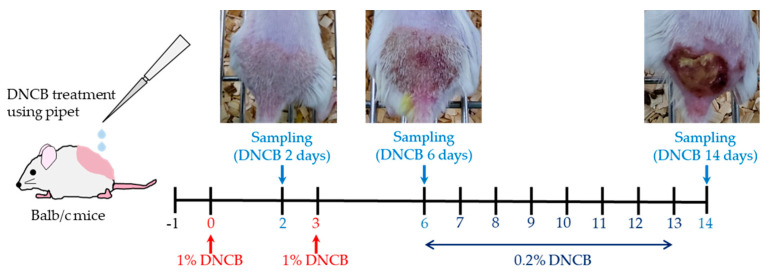
Study design for the induction of atopic dermatitis (AD) in the animal model using 2,4-dinitrochlorobenzene (DNCB). The DNCB 2 days group was induced by one treatment with 1% DNCB. For the DNCB 6 days group, AD was induced by treatment with DNCB once per day on day 0 and day 3. Finally, the DNCB 14 days group was sensitized with 1% DNCB on day 0 and day 3, and then subsequently treated with 2% DNCB daily from day 6 to day 13.

**Figure 2:**
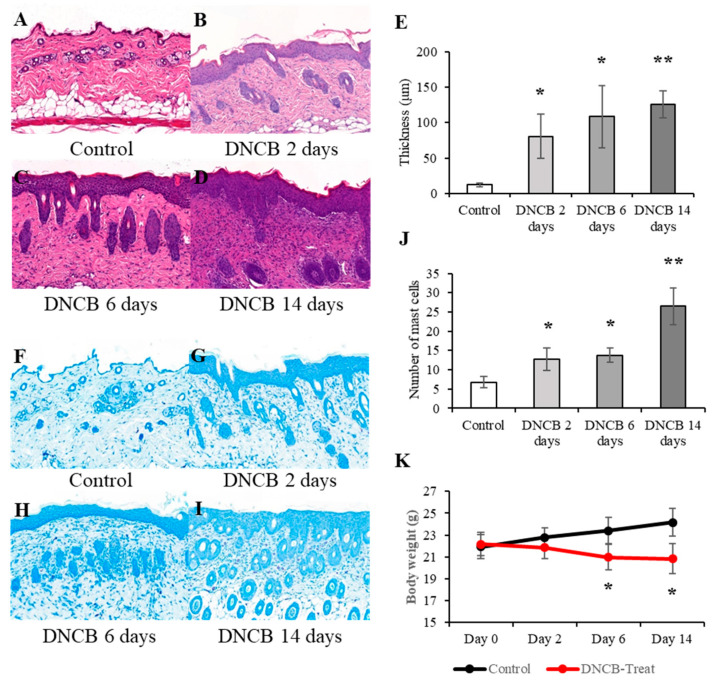
Atopic dermatitis (AD)-like lesions and body weight loss were induced by 2,4-dinitrochlorobenzene. Hematoxylin and eosin staining showed an increase in the stratum corneum and the epidermal thickness, which are typical AD lesions; (**A**) control, (**B**) DNCB 2 days, (**C**) DNCB 6 days, and (**D**) DNCB 14 days. (**E**) Quantification of changes in the thickness of the epidermis. The data are expressed as the mean ± SD; * = *p* < 0.05, ** = *p* < 0.001. Toluidine blue staining was performed to identify mast cell infiltration of the dermis; (**F**) control, (**G**) DNCB 2 days, (**H**) DNCB 6 days, and (**I**) DNCB 14 days. (**J**) Quantification of mast cells infiltrating into the dermal layer. The data are expressed as the mean ± SD; * = *p* < 0.05, ** = *p* < 0.001. (**K**) The change in body weight during DNCD-treatment is shown. The data are expressed as the mean ± SD; * = *p* < 0.05, *n* = 5 per group.

The AD model was generated by treating DNCB with the detailed protocol shown in Figure 1. Then, histopathological analysis of the skin was conducted to determine whether DNCB induced AD. Firstly, an increase in the thickness of the stratum corneum and epidermis—a representative lesion of AD—was evaluated by performing H&E staining (Figure 2A–E; DNCB 2 days = 80.6 ± 31.46, *p* = 0.008; DNCB 6 days = 108.54 ± 43.89, *p* = 0.022; DNCD 14 days = 125.74 ± 19.25, *p* = 0.00016). Next, toluidine blue staining was performed to identify mast cell infiltration of the dermis. The number of infiltrating mast cells increased as the DNCB treatment was repeated (Figure 2F–J; control = 6.75 ± 1.5, DNCB 2 days = 12.75 ± 2.87, *p* = 0.017; DNCB 6 days = 13.75 ± 1.89, *p* = 0.0014; DNCD 14 days = 26.5 ± 4.80, *p* = 0.0022). In addition, body weight loss was observed as the animals were repeatedly treated with DNCB (Figure 2K; day 0 = 22.17 ± 1.06 vs. control (21.92 ± 1.08), *p* = 0.67; day 2 = 21.86 ± 1.05 vs. control (22.78 ± 0.87), *p* = 0.088; day 6 = 20.97 ± 1.15 vs. control (23.41 ± 1.21), *p* = 0.0060; day 14 = 20.82 ± 1.37 vs. control (24.17 ± 1.26), *p* = 0.0039).

### 3.2. Histopathological Analysis of the Hippocampus of Atopic Dermatitis Model Mice

**Figure 3:**
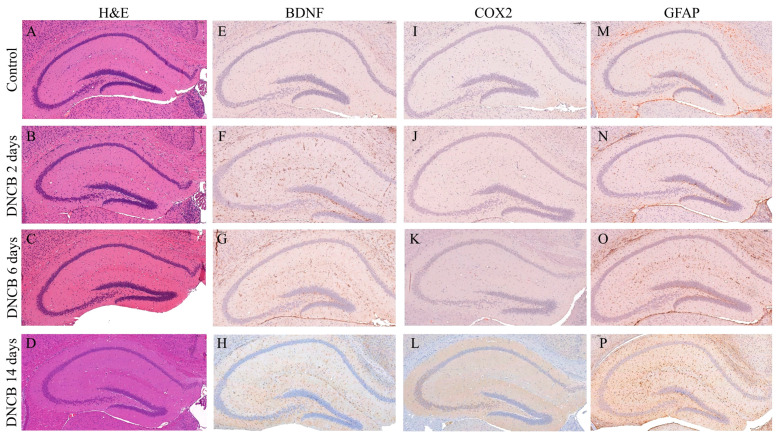
Atopic dermatitis altered the expression patterns of neuroinflammation markers in the hippocampus. Representative hematoxylin and eosin staining images of the hippocampus; (**A**) control, (**B**) DNCB 2 days, (**C**) DNCB 6 days, and (**D**) DNCB 14 days. Immunohistochemical (IHC) staining of brain-derived neurotrophic factor (BDNF) in the hippocampus; (**E**) control, (**F**) DNCB 2 days, (**G**) DNCB 6 days, and (**H**) DNCB 14 days. IHC staining of cyclooxygenase-2 (COX2) in the hippocampus; (**I**) control, (**J**) DNCB 2 days, (**K**) DNCB 6 days, and (**L**) DNCB 14 days. IHC staining of glial fibrillary acidic protein (GFAP) in the hippocampus; (**M**) control, (**N**) DNCB 2 days, (**O**) DNCB 6 days, and (**P**) DNCB 14 days.

**Figure 4:**
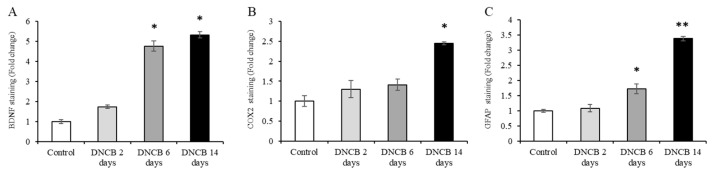
Quantification of atopic dermatitis (AD)-induced changes in the expression of neuroinflammation markers in the hippocampus. Neuroinflammation markers in the hippocampus were altered by AD. The changes in the expressions of brain-derived neurotrophic factor (BDNF), cyclooxygenase-2 (COX2), and glial fibrillary acidic protein (GFAP) were quantified using ImageJ software (NIH, Bethesda, MD, USA). (**A**) BDNF expression changes in the hippocampus. (**B**) COX2 expression changes in the hippocampus. (**C**) GFAP expression changes in the hippocampus. The data are expressed as the mean ± SD; * = *p* < 0.05, ** = *p* < 0.001, *n* = 5 per group.

H&E staining and IHC were performed for analyzing the changes in the neuroinflammation marker expression that was induced by AD. The photomicrographs of the H&E staining showed that there were no significant differences in the hippocampus of the DNCB 2 days, 6 days, or 14 days groups in comparison with the control group (Figure 3A–D). However, quantification of the IHC staining resulted in significant changes in the neuroinflammation markers in the hippocampus (Figure 3E–P). BDNF expression in the hippocampus was observed at significant levels in the DNCB 6 days and 14 days groups (Figure 4A; DNCB 2 days = 1.73 ± 0.083 related to control, *p* = 0.11; DNCB 6 days = 4.76 ± 0.26 related to control, *p* = 0.014; DNCD 14 days = 5.32 ± 0.16 related to control, *p* = 0.0022). In addition, COX2 underwent a significant increase in its expression within the DNCB 14 days group (Figure 4B; DNCB 2 days = 1.30 ± 0.21 related to control, *p* = 0.25; DNCB 6 days = 1.41 ± 0.14 related to control, *p* = 0.13; DNCD 14 days = 2.45 ± 0.033 related to control, *p* = 0.0015). Moreover, GFAP expression levels were significantly altered in the DNCB 6 days and 14 days groups (Figure 4C; DNCB 2 days = 1.08 ± 0.11 related to control, *p* = 0.33; DNCB 6 days = 1.72 ± 0.16 related to control, *p* = 0.010; DNCD 14 days = 3.38 ± 0.077 related to control, *p* = 0.0000055).

### 3.3. Identification of Total Exosomes Isolated from Serum

**Figure 5:**
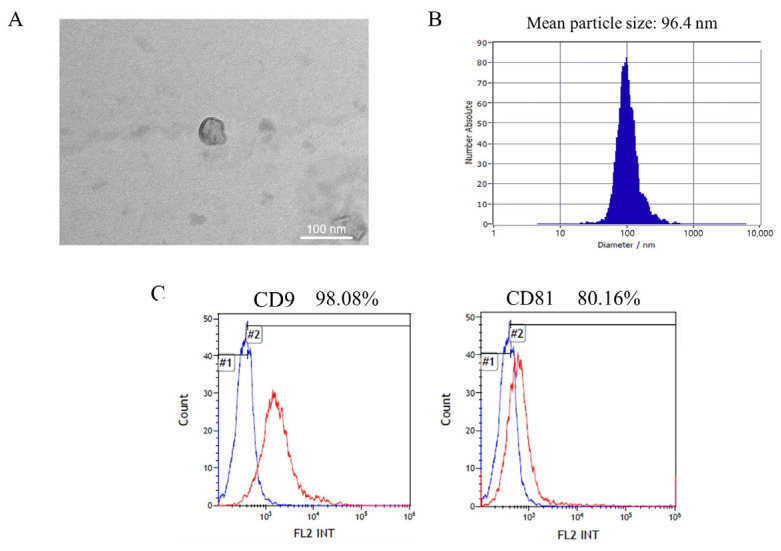
Isolation and characterization of exosomes obtained from serum. (**A**) Transmission electron microscopy showing exosome morphology and size. Scale bar = 100 nm. (**B**) Nanoparticle tracking analysis of exosomes in the mouse serum to confirm the size distribution. (**C**) Flow cytometry data confirming the detection of exosome markers (CD9, CD81) (#1; Negative control, #2; CD9, CD81-positive).

Before the isolation of the nEVs, the tEVs were characterized in several ways. Firstly, TEM was performed to identify the shape and size of the exosomes. Observation of the TEM images demonstrated that exosomes had a spherical bilayer membrane, and were 30–200 nm in diameter (Figure 5A). NTA was used to confirm the size of the exosomes, and the exosome sizes ranged from 30 to 200 nm (Figure 5B). Finally, flow cytometry using the immune-labeling method was performed to identify the presence of exosome markers, such as CD9 and CD81. These results demonstrate that those particles present within the exosomes that were isolated from serum were 98.08% positive for CD9, and 80.16% were CD81-positive (Figure 5C).

### 3.4. Characterization of Isolated Neuronal Exosomes

**Figure 6:**
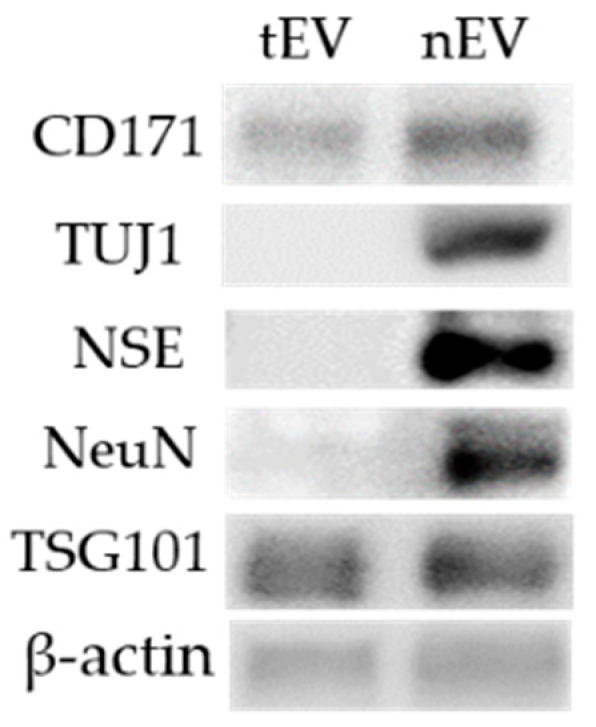
Characterization of neuronal exosomes (nEVs) isolated from mouse serum. Western blot data demonstrating the enrichment of nEV-associated markers. nEV marker; CD171. Neuronal markers; neuron-specific class Ⅲ beta-tubulin (TUJ1), neuron-specific enolase (NSE), and neuronal nuclei (NeuN). Exosome marker; tumor susceptibility gene 101 (TSG101). Western blot was performed in triplicate.

Total exosomes were isolated from the serum, and the separation of the nEVs was conducted using immunoprecipitation with the CD171 biotinylated antibody [31]. Western blot was used to identify the nEVs. These data showed that there was a greater abundance of CD171 in the nEVs than in the tEVs, and neuronal markers, such as TUJ1, NSE, and NeuN, were present in the nEVs, but were not within the tEVs. Finally, in both of the total exosomes and nEVs, TSG101 and β-actin were observed (Figure 6).

### 3.5. Changed Exosomal microRNA Expression Was Induced by Atopic Dermatitis-Induced Stress

**Figure 7:**
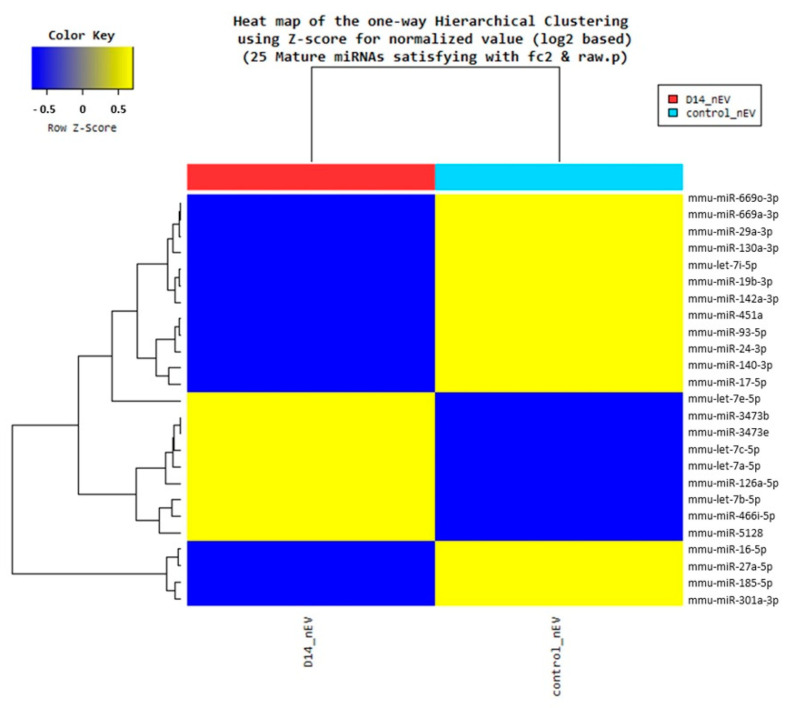
Heatmap of the differential microRNA (miRNA) expression in neuronal exosomes induced by atopic dermatitis. A total of 9 miRNAs were upregulated and 16 miRNAs were downregulated in the DNCB 14 days group compared to the control group.

Any changes in the miRNA expression, as a direct result of AD-induced stress, were confirmed using NGS. The NGS results show that 9 miRNAs were significantly upregulated and 16 were significantly downregulated in the DNCB 14 days group compared with the control group (Figure 7). Those miRNAs that were upregulated in the DNCB 14 days group were mmu-let-7a-5p, mmu-let-7b-5p, mmu-let-7c-5p, mmu-let-7e-5p, mmu-miR-126a-5p, mmu-miR-3473b, mmu-miR-3473e, mmu-miR-466i-5p, and mmu-miR-5128. The downregulated miRNAs were mmu-let-7i-5p, mmu-miR-130a-3p, mmu-miR-140-3p, mmu-miR-142a-3p, mmu-miR-16-5p, mmu-miR-17-5p, mmu-miR-185-5p, mmu-miR-19b-3p, mmu-miR-24-3p, mmu-miR-27a-5p, mmu-miR-29a-3p, mmu-miR-301a-3p, mmu-miR-451a, mmu-miR-669a-3p, mmu-miR-669o-3p, and mmu-miR-93-5p (Table 1). The sequences of these miRNAs are detailed in Supplementary Figure S1.

The target genes of these significantly increased miRNAs were predicted by using mirWalk. The results of target gene prediction are shown in Supplementary Figure S2. Then, GO analysis and KEGG pathway analysis were performed on the genes predicted for the target. GO analysis was performed to confirm which function the predicted target genes was associated with from each of the three categories of biological process (BP), cell component (CC), and molecular function (MF). The 10 most enriched GO terms in each category are shown in Supplementary Figure S3A–C. In addition, the KEGG pathway analysis was performed to confirm which pathway the predicted target genes were associated with. The results are shown in Supplementary Figure S3D.

## 4. Discussion

This study aimed to identify and analyze markers whose expression levels changed due to stress caused by a specific disease, and confirm their potential as stress-related biomarkers. After confirming that neuroinflammation was induced in AD animal models, nEVs were isolated from the sera of the AD models, and the expression levels of exosomal miRNAs were compared with the control group.

The AD animal model that expresses AD-like symptoms was induced with DNCB treatment. DNCB is a substance that causes contact dermatitis through increasing serum immunoglobulin E (IgE) or Th2 cytokine levels (IL-4, IL-13, etc.) [33]. The stress response is very similar to the response to AD. Additionally, AD-like skin lesions occur in mice treated with DNCB due to chemical irritation; therefore, DNCB is widely used when developing AD animal models [33]. In this study, we confirmed that DNCB did indeed induce AD-like lesions, including increased stratum corneum, epidermal thickness, and increased mast cell infiltration of the dermis. These results have been confirmed in an AD model [33,34]. It was also shown that increased DNCB treatment times resulted in poorer symptomatology. In addition, previous studies have demonstrated the correlation between AD and the HPA axis [35,36]. According to earlier studies, the concentrations of adrenocorticotropic hormone (ACTH) and corticosterone in the plasma increased in the AD animals in comparison to the control animals [37]. Therefore, it has been shown that the AD animal model induced by DNCB treatment can be used for stress-related studies.

Next, to confirm whether the central nervous system was affected by AD-induced stress, the expression patterns of neuroinflammation-related markers in the hippocampus were analyzed. In the AD animal models, a gradual upregulation in the expressions of BDNF, COX2, and GFAP was observed, which depended on the length of DNCB treatment time. BDNF is highly expressed in the brain, associated with neuronal survival, neuronal maintenance, synaptic plasticity, etc., and is widely known to be associated with neuroinflammation and psychiatric disorders [38,39]. BDNF is also related to the sensory nervous system and plays an important role in experiences of itchiness in AD [40]. While several studies have shown BDNF decreases in major depressive disorder or psychological stress states, some studies have demonstrated plasma or serum BDNF increases in patients with AD [41,42]. The expression pattern of BDNF in the hippocampus will differ depending on the type of stress, and therefore, further research is required.

COX2 and GFAP are known inflammatory markers for neuroinflammation. COX2 is an enzyme that is closely related to the induction of pain and inflammation [43]. In lipopolysaccharide-induced neuroinflammation, COX2 is upregulated [44]. Other studies have demonstrated that the suppression of COX2 reduces the symptoms of stress-related psychiatric disorders [45]. GFAP is expressed in astrocytes, and enables the protection and activity of the neuron, promoting synaptic plasticity [46]. In addition, many studies have shown that this marker is upregulated in the hippocampus of the neuroinflammation models [47,48].

Exosomes have been evaluated for their potential as biomarkers for various diseases. Additionally, there are reports that psychological stress can affect the brain and cause neuroinflammation. Because of this, nEVs were isolated from tEVs in serum to investigate stress-related markers. When separating nEVs, the surface marker CD171 (L1CAM)—also known as an nEV surface marker—was used [32]. CD171, a neural adhesion protein, is involved in the development of the nervous system [49]. The neuron-related markers TUJ1, NSE, and NeuN were also confirmed in this study [50,51,52]. Immunoprecipitation against CD171 has previously been used to isolate nEVs and confirm that they contain more neuron-related markers than tEVs [32].

Bioinformatics is frequently used to identify the constituents in exosomes, which is important for the detection of potential biomarkers. For instance, exosomal proteins are analyzed using proteomics, and NGS is widely used for exosomal miRNAs analysis in a diverse number of cancers [53,54,55]. In this study, the expression patterns of miRNAs in nEVs were altered when AD was induced, as confirmed using NGS. Using the miRDB database (http://miRDB.org/; accessed on 31 August 2021; Xiaowei Wang’s lab at the Department of Radiation Oncology, Washington University School of Medicine in St. Louis, United States), it was observed that these miRNAs target several genes, some of which are associated with neurons, stress, and AD. For example, the let-7a-5p target chemokine receptor CCR7 is related to the immune cells in AD lesions [56]. A decrease in proliferation occurred with the presence of let-7b in the neural stem cells, and this also increased neural differentiation [57]. Furthermore, the expression of let-7c-5p decreased in a traumatic brain injury (TBI) model, and the overexpression of this miRNA attenuates neuroinflammation after TBI induction [58]. In addition, let-7a-5p, let-7c-5p, and let-7i-5p are all upregulated in acute social defeat stress and chronic unpredictable mild stress (CUMS)-induced depression, with let-7b-5p upregulated and miR-140-3p downregulated in CUMS-induced depression [59,60]. Therefore, these results suggest that the expression patterns of miRNAs in nEVs were altered by stress, and these miRNAs could be used as stress-related markers.

## 5. Conclusions

In this study, AD was successfully induced, nEVs were isolated from serum, and the changes in the exosomal miRNA expressions were evaluated. In conclusion, the potential use of miRNAs in nEVs as stress-specific markers was confirmed. However, neuronal exosomal miRNA expression patterns differ depending on the type of stressor and disease, and the target genes of significantly increased miRNAs are numerous and have diverse functions. Therefore, further studies must evaluate other stressors and validate these stress-specific biomarker candidates.

## Figures and Tables

**Table 1:** Up/downregulation of microRNAs (miRNAs) induced by atopic dermatitis.

Mature MiRNA	Predicted Target Genes	Fold Change (Severe/Control)	MiRNA Expression in Atopic Dermatitis
mmu-let-7a-5p	865	3.32	Upregulation
mmu-let-7b-5p	867	1.89	Upregulation
mmu-let-7c-5p	865	2.67	Upregulation
mmu-let-7e-5p	865	24.05	Upregulation
mmu-miR-126a-5p	953	2.33	Upregulation
mmu-miR-3473b	613	1.71	Upregulation
mmu-miR-3473e	613	1.75	Upregulation
mmu-miR-466i-5p	1934	1.93	Upregulation
mmu-miR-5128	153	3.51	Upregulation
mmu-let-7i-5p	870	0.02	Downregulation
mmu-miR-130a-3p	693	0.03	Downregulation
mmu-miR-140-3p	681	0.1	Downregulation
mmu-miR-142a-3p	374	0.03	Downregulation
mmu-miR-16-5p	1175	0	Downregulation
mmu-miR-17-5p	966	0.03	Downregulation
mmu-miR-185-5p	987	0	Downregulation
mmu-miR-19b-3p	1027	0.02	Downregulation
mmu-miR-24-3p	966	0.09	Downregulation
mmu-miR-27a-5p	202	0	Downregulation
mmu-miR-29a-3p	862	0.05	Downregulation
mmu-miR-301a-3p	688	0	Downregulation
mmu-miR-451a	42	0.07	Downregulation
mmu-miR-669a-3p	373	0.06	Downregulation
mmu-miR-669o-3p	373	0.06	Downregulation
mmu-miR-93-5p	963	0.07	Downregulation

## Data Availability

The data that support the findings of this study are available from the corresponding author upon reasonable request.

## Supplementary Materials

The following are available online at https://www.mdpi.com/article/10.3390/biomedicines9121764/s1, Figure S1: MicroRNA (miRNA) sequences that were differentially expressed following atopic dermatitis. Figure S2: The target genes of significantly increased miRNAs predicted by using mirWalk. Figure S3: GO analysis and KEGG pathway analysis indicating that neuronal exosomal miRNAs with different expression patterns under atopic dermatitis.
