# Supplementary material for: Serum-Derived Neuronal Exosomal microRNAs as Stress-Related Biomarkers in an Atopic Dermatitis Model
Source: Biomedicines. 2021 Nov 25;9(12):1764. doi: 10.3390/biomedicines9121764 (PMC8698818; doi:10.3390/biomedicines9121764)
Supplement: Supplementary file 1 [file biomedicines-09-01764-s001.zip › biomedicines-1445483-supplementary/Supplementary Figure S1.pdf]

Supplementary Material S1. MicroRNA (miRNA) sequences that were differentially expressed following atopic dermatitis.

Mature miRNA	Sequence (5′-3′)
mmu-let-7a-5p	UGAGGUAGUAGGUUGUAUAGUU
mmu-let-7b-5p	UGAGGUAGUAGGUUGUGUGGUU
mmu-let-7c-5p	UGAGGUAGUAGGUUGUAUGGUU
mmu-let-7e-5p	UGAGGUAGGAGGUUGUAUAGUU
mmu-miR-126a-5p	CAUUAUUACUUUUGGUACGCG
mmu-miR-3473b	GGGCUGGAGAGAUGGCUCAG
mmu-miR-3473e	GGGCUGGAGAGAUGGCUCGUA
mmu-miR-466i-5p	UGUGUGUGUGUGUGUGUGUG
mmu-miR-5128	CAAUUGGGGCUGGCGAGAUGGCU
mmu-let-7i-5p	UGAGGUAGUAGUUUGUGCUGUU
mmu-miR-130a-3p	CAGUGCAAUGUUAAAAGGGCAU
mmu-miR-140-3p	UACCACAGGGUAGAACCACGG
mmu-miR-142a-3p	UGUAGUGUUUCCUACUUUAUGGA
mmu-miR-16-5p	UAGCAGCACGUAAAUAUUGGCG
mmu-miR-17-5p	CAAAGUGCUUACAGUGCAGGUAG
mmu-miR-185-5p	UGGAGAGAAAGGCAGUUCCUGA
mmu-miR-19b-3p	UGUGCAAAUCCAUGCAAAACUGA
mmu-miR-24-3p	UGGCUCAGUUCAGCAGGAACAG
mmu-miR-27a-5p	AGGGCUUAGCUGCUUGUGAGCA
mmu-miR-29a-3p	UAGCACCAUCUGAAAUCGGUUA
mmu-miR-301a-3p	CAGUGCAAUAGUAUUGUCAAAGC
mmu-miR-451a	AAACCGUUACCAUUACUGAGUU
mmu-miR-669s-3p	ACAUAACAUACACACACACGUAU
mmu-miR-669o-3p	ACAUAACAUACACACACACGUAU
mmu-miR-93-5p	CAAAGUGCUGUUCGUGCAGGUAG
