# Supplementary material for: Serum-Derived Neuronal Exosomal microRNAs as Stress-Related Biomarkers in an Atopic Dermatitis Model
Source: Biomedicines. 2021 Nov 25;9(12):1764. doi: 10.3390/biomedicines9121764 (PMC8698818; doi:10.3390/biomedicines9121764)

Supplementary Figure S3. Gene ontology (GO) analysis and Kyoto Encyclopedia of Genes and Genomes (KEGG) pathway analysis indicating that neuronal exosomal miRNAs with different expression patterns under atopic dermatitis.

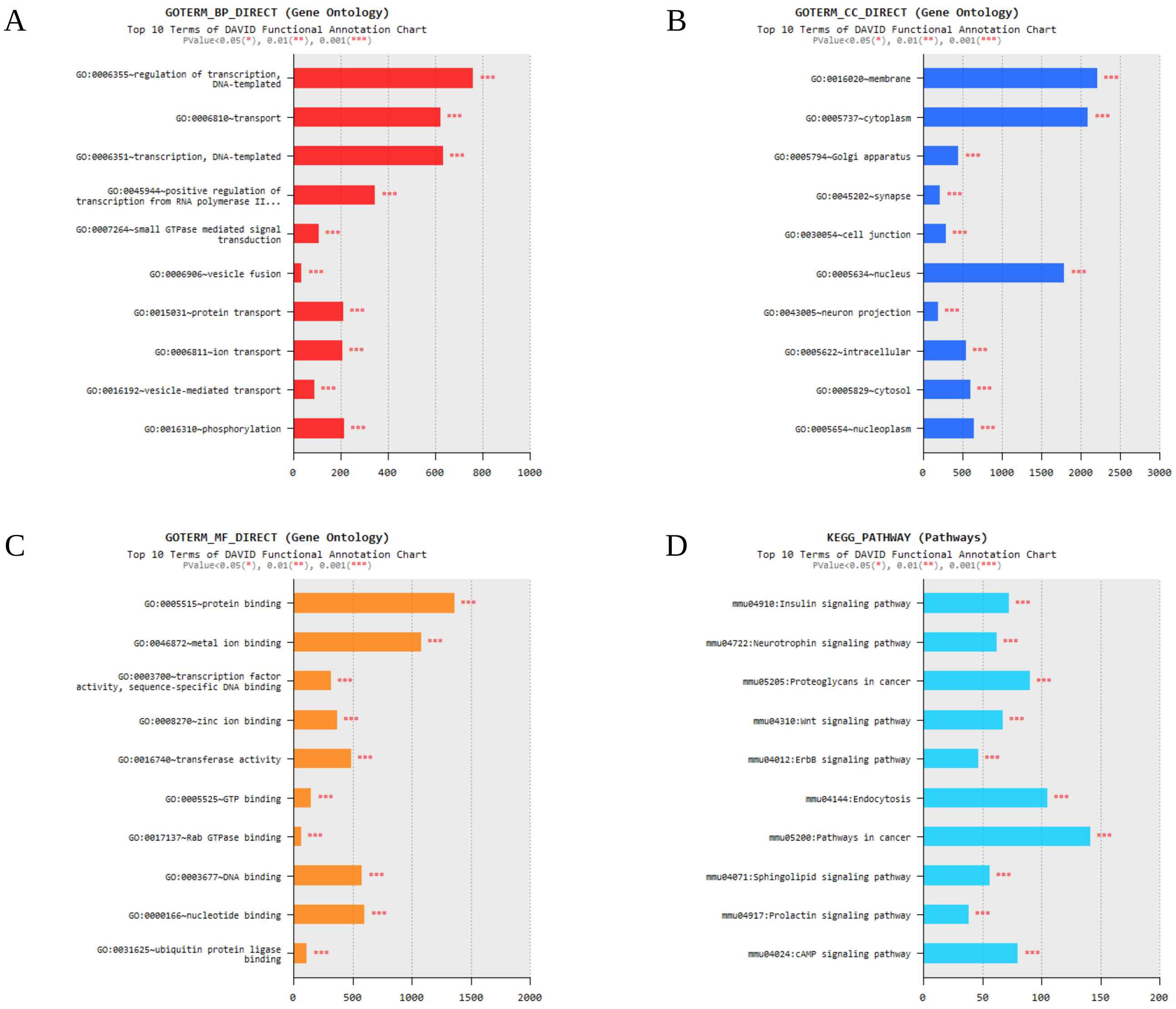

Supplement: Supplementary file 1 [file biomedicines-09-01764-s001.zip › biomedicines-1445483-supplementary/Supplementary Figure S3.pdf]
